# P02-030 - Unusual CNS manifestation

**DOI:** 10.1186/1546-0096-11-S1-A137

**Published:** 2013-11-08

**Authors:** E Schuh, P Lohse, I Meinl, T Kümpfel

**Affiliations:** 1 Neurology, Institute of Clinical Neuroimmunology, Munich, Germany; 2 Laboratoriumsmedizin, Labor Blessing, Singen, Germany

## Introduction

Cryopyrin-associated periodic syndrome (CAPS) is a rare systemic, monogenetic inherited autoinflammatory condition caused by NLRP3/CIAS 1 gene mutations encoding for cryopyrin, a major component of the inflammasome, leading to an excessive production of interleukin-1beta (IL-1ß). The clinical picture of genetic variations of the NLRP3 inflammasome is characterized by recurrent episodes of systemic inflammation involving skin, joints, eyes and the central nervous system and shows variable penetrance regarding disease severity and symptoms.

## Case report

Here, we report an unusual case of a 43 year-old woman who presented to us with diagnosis of CRION (chronic relapsing inflammatory optic neuropathy) due to recurrent episodes of optic neuritis on both eyes leading to atrophy of the left optical nerve with blindness. Repeated cerebral MRI scan showed an unusual excessive contrast enhancement involving both optic nerves with massive swelling as well as contrast enhancement in the oculomotor nerve and the pituitary gland. Additionally a few unspecific hyperintense white matter lesions were seen. Examination of the cerebrospinal fluid (CSF) revealed a mild pleocytosis and oligoclonal bands. Extensive laboratory investigations for other inflammatory CNS diseases as well as rheumatological diseases remained all unremarkable. Treatment with high dose i.v. steroids improved visual acuity, but worsened again after cessation of steroid therapy.

Furthermore the patient reported about intermittent tendinitis and tension type headache. Laboratory examinations revealed recurrent increased levels of Serum Amyloid A (SAA) and C-reactive protein (CRP). Molecular genetic testing showed a homozygous Q703K mutation in Exon 3 of the NLRP3/CIAS 1 gene. Diagnosis of CAPS was set and high dose anti-Interleukin 1 therapy initiated which led so far to moderate improvement of the CNS inflammation as well as reduction of SAA levels. The patient is currently followed up.

## Discussion

This is an unusual and severe CNS manifestation in a patient homozygous for the Q703K variant in exon 3 of the NLRP3/CIAS 1 gene which has been considered so far as a low-penetrance mutation with a mild phenotype. Ocular manifestations including optic nerve atrophy usually occur in classical CICNA/NOMID patients. This case illustrates that CAPS should be also considered in adult patients with chronic recurrent optic neuritis and additional symptoms suggestive of an autoinflammatory syndrome.

## Disclosure of interest

E. Schuh: None Declared, P. Lohse: None Declared, I. Meinl: None Declared, T. Kümpfel Grant / Research Support from: Novartis.
